# Overexpression of MicroRNA-200c Predicts Poor Outcome in Patients with PR-Negative Breast Cancer

**DOI:** 10.1371/journal.pone.0109508

**Published:** 2014-10-16

**Authors:** Marie Tuomarila, Kaisa Luostari, Ylermi Soini, Vesa Kataja, Veli-Matti Kosma, Arto Mannermaa

**Affiliations:** 1 Institute of Clinical Medicine, Clinical Pathology and Forensic Medicine, University of Eastern Finland, Kuopio, Finland; 2 Institute of Clinical Medicine, Oncology, University of Eastern Finland, Kuopio, Finland; Sanjay Gandhi Medical Institute, India

## Abstract

Micro-RNAs are small, noncoding RNAs that act as tumor suppressors or oncogenes. MiR-200c is a member of the miR-200 family; it is known to be dysregulated in invasive breast carcinoma. MiR-200c maintains the epithelial-mesenchymal transition and inhibits cell migration and invasion. Recent studies showed that miR-200c regulated steroid hormone receptors, estrogen receptors (ER), and progesterone receptors (PR). The present study aimed to detect miR-200c in 172 invasive breast carcinoma cases selected from a prospective cohort enrolled in Kuopio, Eastern Finland, between 1990 and 1995. MiR-200c expression was determined with relative q-PCR, and results were compared to clinicopathological variables and patient outcome. We found that PR status combined with miR-200c expression was a significant marker of outcome. High miR-200c expression was associated with reduced survival in PR-negative cases (*n* = 68); low miR-200c expression indicated reduced survival in PR-positive cases (*n* = 86) (Cox regression: *P* = 0.002, OR = 3.433; and *P* = 0.004, OR = 4.176, respectively). In PR-negative cases, high miR-200c expression was associated with shortened relapse-free survival (Cox regression: *P* = 0.001, OR = 3.613); increased local/distant recurrence (Logistic regression: *P* = 0.006, OR = 3.965); and more frequent distant metastasis (Logistic regression: *P* = 0.015, OR = 3.390). We also found that high grade and low stage tumors were positively correlated with high miR-200c expression (Logistic regression for high grade tumors: *P* = 0.002, OR = 2.791 and for high stage tumors: *P* = 0.035, OR = 0.285). Our results indicated that miR-200c may play a role in invasive breast carcinoma. Furthermore, miR-200c combined with PR status provided a refined predictor of outcome. In future, a larger study is required to confirm our results. This data may provide a basis for new research target–progesterone receptor–regulated microRNAs in breast cancer.

## Introduction

Breast cancer is a heterogeneous disease with subtypes characterized by many different biological and clinical features. The disease prognosis varies with the subtype and other clinical markers [1]–[3]. Immunohistochemical studies are routinely conducted to detect molecular markers that identify different subtypes. These markers include the estrogen receptor (ER), progesterone receptor (PR), human epidermal growth factor receptor 2 (*Her2*), and Ki-67 labeling index. The detection of these markers facilitates diagnosing and planning the appropriate therapy [4]. Nevertheless, novel diagnostic methods and better factors for predicting risk are needed to extend our understanding of breast cancer and to develop new therapeutic methods for combating cancer.

Hormonal functions in breast cancer and normal breast tissue have been widely studied. Evidence emphasizes the role of both estrogen and progesterone steroid hormones in the initiation and growth of breast tumors. The actions of progesterone and estrogen in breast cancer are mediated by their cognate receptors, PR and ER, respectively. Recent studies have suggested that there may be a link between deregulated microRNAs (miRNAs) and steroid hormones in malignancies [5], [6].

miRNAs are small, non-coding RNAs, approximately 19–24 nucleotides long, which bind specifically to the 3′-untranslated regions (3′-UTRs) of target mRNAs and regulate their translation to proteins [7]. In recent years, studies have revealed an association between the differential expression of microRNAs and cancerous tissues. The miRNAs involved in cancer may act as tumor suppressors or oncogenes, depending on the target [8]–[10].

The miR-200 family includes the mature miR-200c, miR-141, miR-200b, miR-200a, and miR-429 [11]. These miRNAs have been shown to maintain the epithelial phenotype in breast cancer; to inhibit cell migration and invasion by targeting epithelial-mesenchymal transition (EMT) repressors and transcriptional factors ZEB1 and ZEB2 [12]–[15]; to suppress the genes involved in migration, such as *MSN*, *FN1* [16], and *WAVE3* [17]; and to directly target actin-regulatory proteins, FHOD1 and PPM1F [18]. MiR-200c restores anoikis sensitivity by targeting TrkB [16], and it regulates induction of apoptosis through CD95 by targeting *FAP-1* [19]. The miR-200 family was found to be differentially expressed in different breast cancer subtypes [20], [21]. Its expression was downregulated in metastatic breast cancer [22]. Moreover, DNA methylation is a major epigenetic mechanism that regulates the transcriptional activation of the miR-200c/141 gene cluster. Different breast cancer subtypes have shown differential levels of genomic DNA methylation [22].

Recently, researchers have shown an increased interest in defining hormone receptor-regulated miRNAs. miRNA signatures may predict the ER, PR and *Her2*-status of patients with breast cancer. Thus, alterations in miRNA expression may be related to changes in hormone receptor status [6]. The ability of ERα to regulate miRNAs in breast cancer has been intensely investigated [23]–[26]. In contrast, few studies have found mechanisms that point to a role for PR in regulating or modulating miRNAs in breast cancer [27]–[31].

In the present study, we aimed to examine the association between miR-200c expression and breast cancer outcome. We examined 172 clinical samples from a cohort of patients from Eastern Finland with invasive breast cancer. The expression of miR-200c and several clinicopathological variables were assessed and analyzed for associations with breast cancer outcome. Our findings revealed that the miR-200c expression level was associated with different outcomes and relapse-free survival rates in patients with PR-positive and PR-negative cancers.

## Materials and Methods

### Materials

There were a total of 172 invasive breast cancer cases available for the present study. The material was unselected and no power calculations were conducted. The case records and samples were acquired from the Kuopio Breast Cancer Project (KBCP), which was conducted from April 1990 to December 1995. More detailed information about the samples and clinical features of the patients in the KBCP was published previously [32], [33].

In this study, the mean age of the 172 patients at the time of diagnosis was 60.4 years. At the cut-off point in February 2011, the average follow-up time was 9.7 years, with a range of 0.10 to 19.8 years. Sixty patients had died of breast cancer during the follow-up, 59 died from other reasons, and 53 patients remained alive. The next cut-off point in July 2013 clarified the metastases of the patients; 60 patients had primary distant metastasis, and 30 patients had secondary, tertiary, or quaternary metastases. The patient and tumor characteristics of the data are summarized in Table 1 and in table S1.

**Table 1:** Clinical characteristics associated with miR-200c expression in patients with breast cancer.

		miR-200c expression^a^			
Clinical variable	*n*	Low expression (%)	High expression (%)	*P*	OR (95% CL)
Age at diagnosis				0.17^b^	
< = 59	85	38 (22.1)	47 (27.3)		
> = 60	87	48 (27.9)	39 (22.7)		
Patient status				0.242^b^	
Dead, breast cancer	60	29 (16.9)	31 (18.0)		
Dead, other cause	59	35 (20.3)	24 (14.0)		
Alive, no recurrence	46	20 (11.6)	26 (15.1)		
Alive, recurrence	7	2 (1.2)	5 (2.9)		
Histological grade				0.002^b^	
I and II	106	63 (37.8)	43 (27.5)		Ref.
III	61	21 (12.6)	40 (24.0)	0.002	2.79 (1.45–5.37)
Histological stage				0.045	
I	44	21 (12.7)	23 (13.9)		Ref.
II	100	45 (27.2)	55 (33.3)	0.762	1.116 (0.548–2.272)
III and IV	21	16 (9.7)	5 (3.0)	0.035	0.285 (0.089–0.915)
Histological type				0.254^b^	
Ductal	117	54 (32.3)	63 (37.7)		
Lobular	31	19 (11.4)	12 (7.2)		
Other	19	11 (6.6)	8 (4.8)		
Estrogen receptor				0.866^b^	
Negative	51	26 (15.7)	25 (15.0)		
Positive	115	57 (34.3)	58 (34.9)		
Progesterone receptor				0.21^b^	
Negative	72	40 (24.1)	32 (19.3)		
Positive	94	^43 (25.9)^	^51 (30.7)^		
*Her2*-status				0.452^b^	
Negative	133	67 (42.1)	66 (41.5)		
Positive	26	^11 (6.9)^	^15 (9.4)^		
Triple negativity				0.541^b^	
Yes	29	^16 (9.3)^	^13 (7.6)^		
No	143	^70 (40.7)^	^73 (42.4)^		
Local/distant recurrence				0.185^b^	
Yes	73	^32 (18.7)^	^41 (24.0)^		
No	98	^53 (31.0)^	^45 (26.3)^		
Primary distant metastasis				0.337^b^	
Yes	60	^27 (15.7)^	^33 (19.2)^		
No	112	^59 (34.3)^	^53 (30.8)^		
Luminal type A/B				0.531^b^	
Luminal A (er+, *Her2*−, pr+)	100	^49 (45.4)^	^51 (47.2)^		
Luminal B (er+, *Her2*+, pr−)	8	^3 (2.8)^	^5 (46.3)^		

Abbreviations: *n*, number of cases; Ref, reference category in the logistic regression analysis.

a Low and high relative expression of miR-200c according to the median value.

b P value assessed by chi-square test; other P values based on the logistic regression analysis.

This study was approved by the Joint Ethics Committee of the University of Kuopio/University of Eastern Finland and the Kuopio University Hospital (written consents 1/1989 and 61/2010). Each patient gave informed written consent for participation in the study.

### Nucleic Acid Isolation and real-time qRT-PCR

RNA was extracted from fresh-frozen tissues stored at −70°C with the *MirVana* miRNA Isolation Kit (Ambion, Austin, Texas, TX). Approximately 30 mg of fresh frozen tissue was taken and total RNA was isolated according to the manufacturer’s protocol.

Complementary DNA (cDNA) was synthesized from 10 ng of total RNA by reverse transcription with the TaqMan MicroRNA Reverse Transcription Kit (Life Technologies, Grand Islands, NY) using gene-specific primers for hsa-miR-200c (ID 002300) and RNU48 (ID 001006), according to the manufacturer’s instructions. We evaluated miRNA levels with TaqMan MicroRNA Assay for miR-200c (ID 002300) and RNU48 (ID 001006) with qRT-PCR following the protocol provided by the manufacturer. The reactions were carried on a Mx3000 Real-Time PCR System (Life Technologies, Grand Islands, NY). The RNU48 primer was used to produce the endogenous control, based on the manufacturer’s recommendations. The standard curves for both miR-200c and RNU48 were generated and used for validation. The relative gene expression values were calculated according to the ΔΔCt method, where the mean threshold cycle value (Ct) for three replicates was used for each sample. The relative expression values (fold change) can be seen in table S2.

Then, the miR-200c expression levels observed in different samples were assigned to one of two groups for further analyses: (1) the moderate/high expression group comprised samples with expression levels greater than median miR-200c level; (2) the low expression group comprised samples with expression levels lower than the median miR-200c level.

### Statistical analysis

The statistical analyses were performed with SPSS for Windows, version 19.0 (SPSS Inc., Chicago, IL, USA). The chi-squared test and the logistic regression analysis were used to compare the miR-200c expression levels among different groups and to determine the association between miRNA-200c expression and clinical variables. Survival rates were evaluated with the Kaplan-Meier method. We used the log-rank, Breslow and Tarone-Ware test, with death due to breast cancer as the end point. To study the association between miR-200c expression and breast cancer survival, Cox regression was used in a multivariate analysis. P-values less than 0.05 were considered statistically significant.

## Results

### High miR-200c expression correlates with grade 3 tumors

First, the relative miR-200c expression rates were compared with the clinicopathological variables of the 172 invasive breast carcinoma samples. Notably, high miR-200c expression was associated with grade 3 tumors (*P* = 0.002, Table 1) and with low stage cancers (*P* = 0.045, Table 1).

### Low miR-200c expression in PR-positive cases is independently associated with poor survival

**Figure 1:**
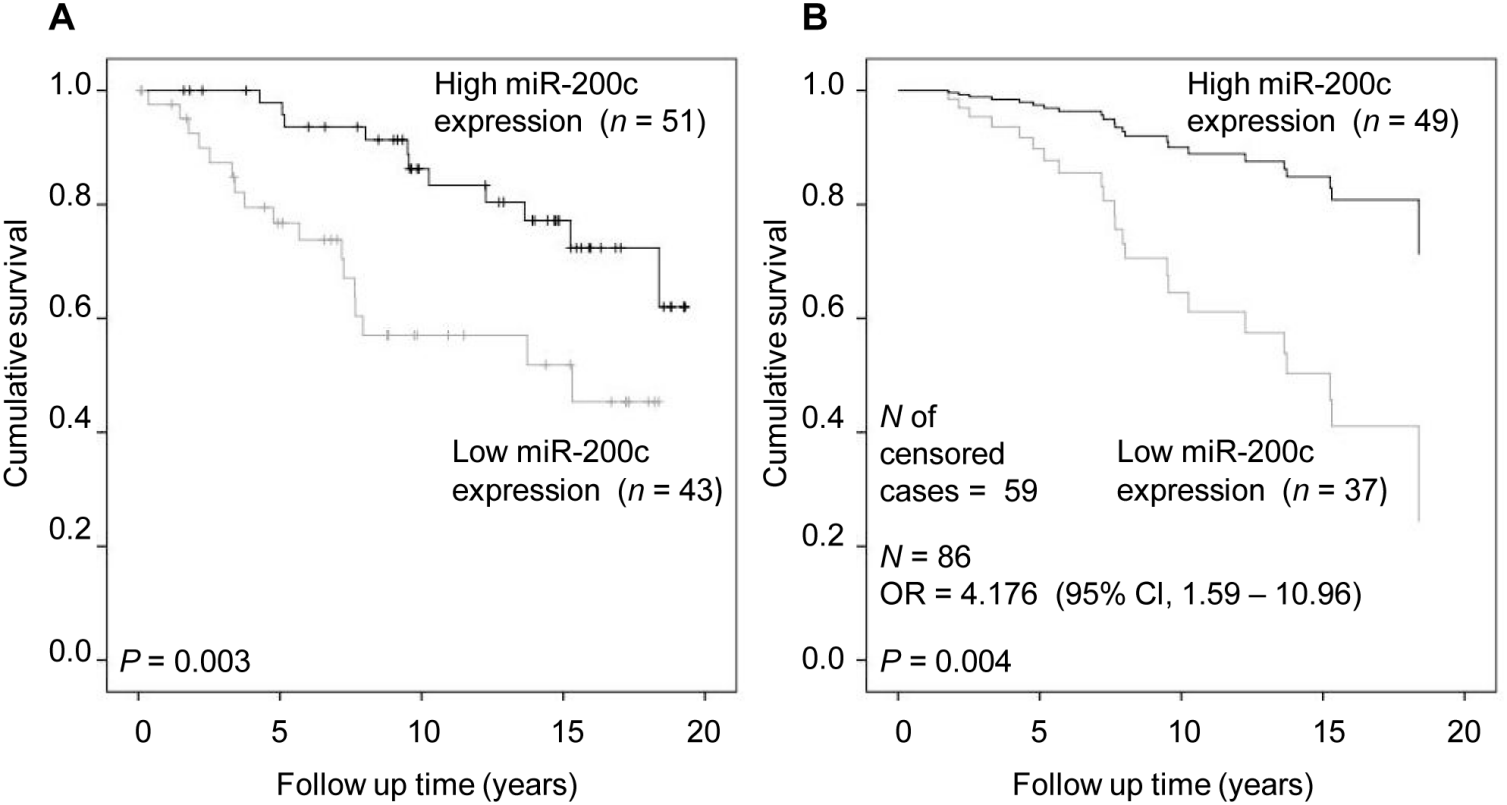
MiR-200c expression and breast cancer specific survival in PR – positive cancer cases. A) Kaplan-Meier analysis; B) Cox regression analysis including covariates age at diagnosis, nodal status, size of the tumor, histological type, *Her2*-status, and ER-status. OR (95% Cl), OR of breast cancer specific death with 95% Cl in multivariate analysis.

When only the patients with PR-positive tumors (*n* = 94) were evaluated with the Kaplan- Meier analysis, low miR-200c expression appeared to predict poor breast cancer specific survival (*P* = 0.003, Figure 1A). Multivariate analysis also showed that low miR-200c expression was an independent factor for predicting poor survival (Figure 1B), in addition to nodal status and *Her2*-status, (*P* = 0.004, OR = 4.176, table S3). The same association was found in both PR and ER positive cancers (*n* = 88) in Kaplan-Meier analysis (*P* = 0.013, figure S1).

### High miR-200c expression in the PR-negative cases predicts poor survival and a short relapse-free survival

**Figure 2:**
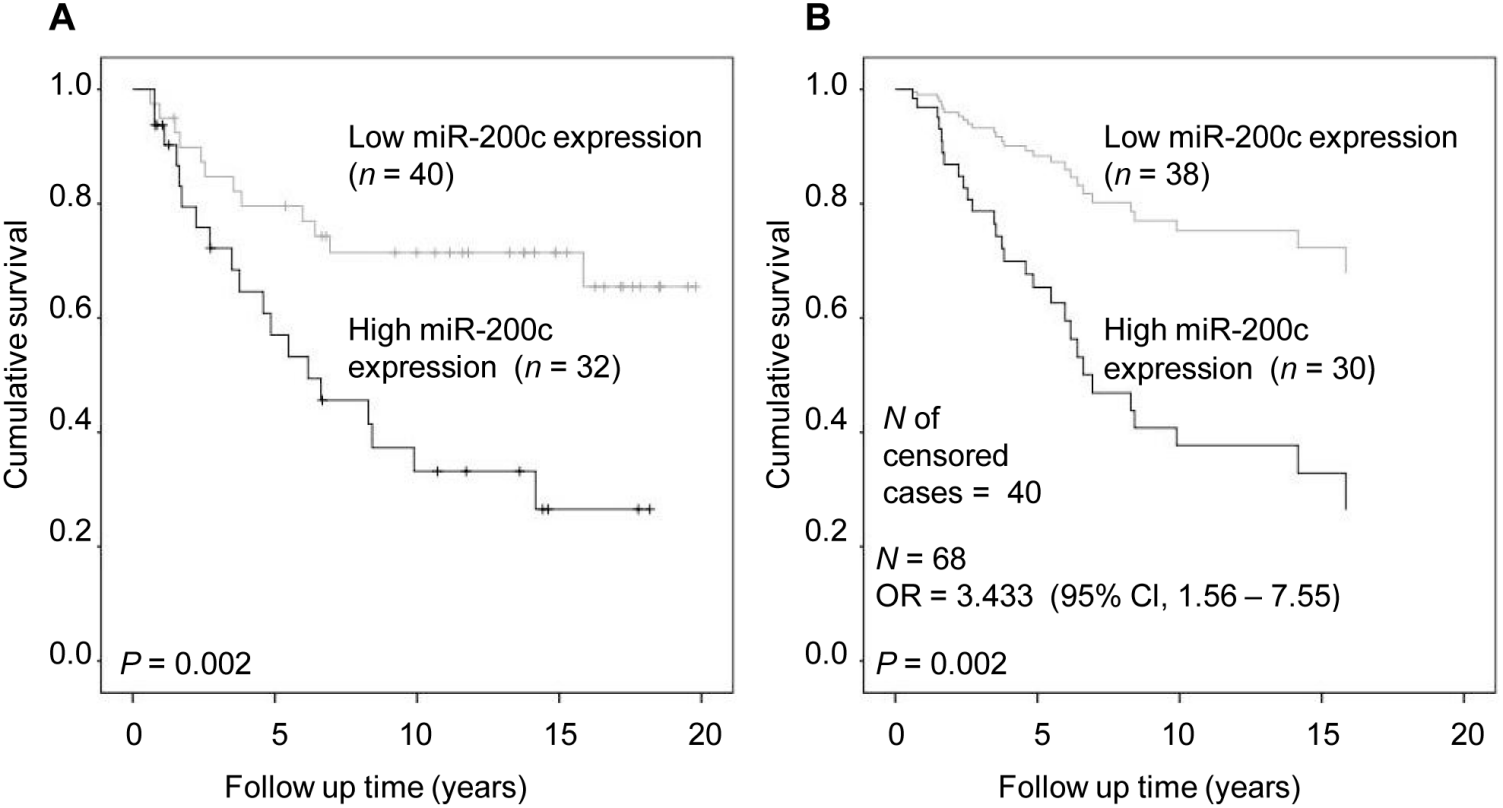
MiR-200c expression and breast cancer specific survival in PR – negative cancer cases. A) Kaplan-Meier analysis; B) Cox regression analysis including covariates age at diagnosis, nodal status, size of the tumor, histological type, *Her2*-status, and ER-status. OR (95% Cl), OR of breast cancer specific death with 95% Cl in multivariate analysis.

When patients with PR-negative tumors (*n* = 72) were evaluated with the Kaplan-Meier analysis, high miR-200c expression appeared to be associated with poor breast cancer specific survival (*P* = 0.002, Figure 2A). The same result was obtained in the multivariate analysis (*P = *0.002, OR = 3.433, Figure 2B). In addition, the histological type, ER status, and tumor size were significantly related to survival (table S4).

Of the 72 PR-negative cases, 45 were both PR-negative and ER-negative. To investigate whether the ER status affected the results, the Kaplan-Meier survival analysis was applied to cases with both PR-negativity and ER-negativity. We found that high miR-200c expression remained associated with poor survival in this group (*P*  = 0.025, figure S2). Interestingly, the Cox analysis did not show a significant association between miR-200c and survival in the group with both ER – and PR – negative cancer in contrast to the Cox analysis of only PR – negative tumors.

**Figure 3:**
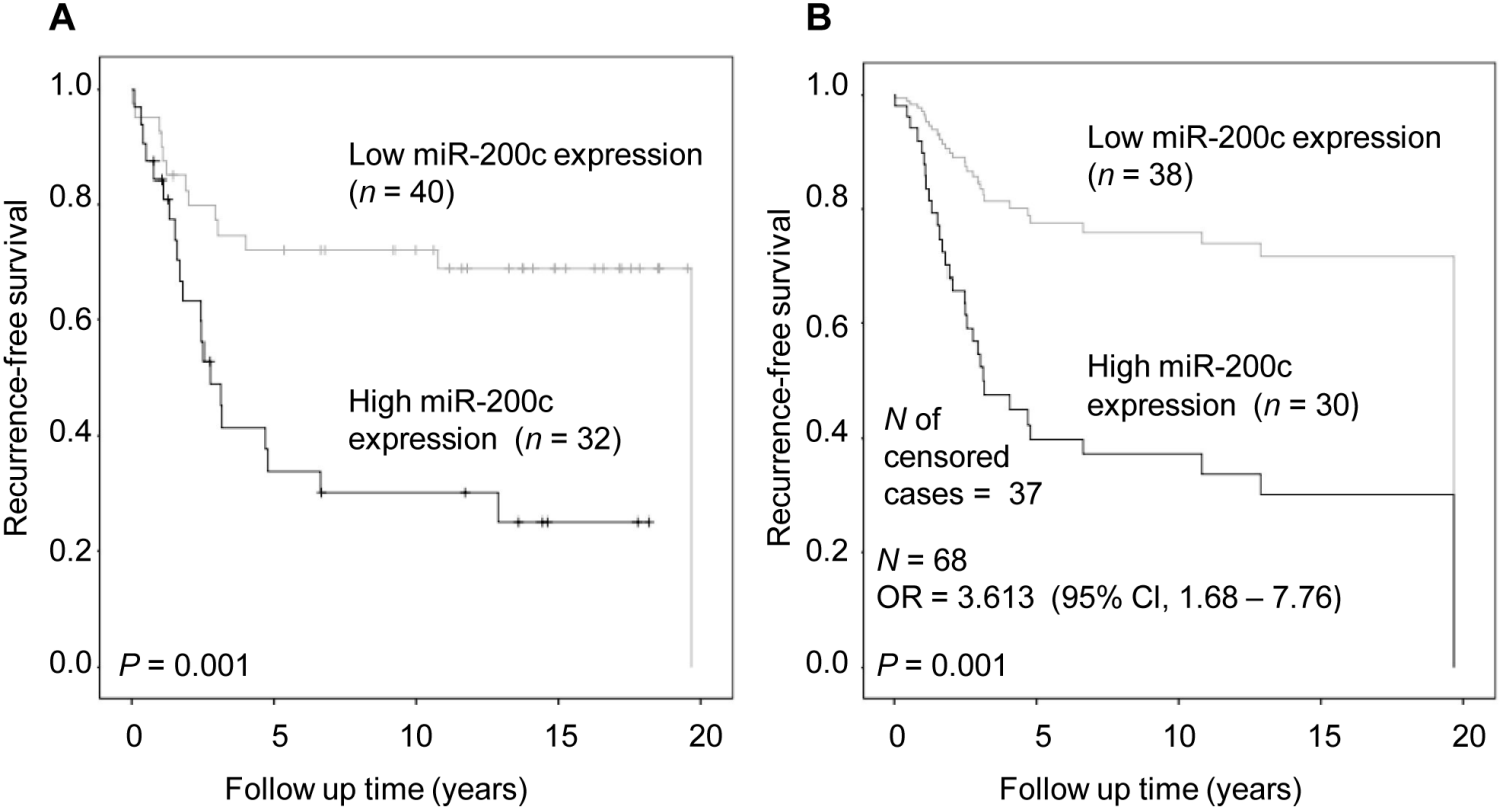
MiR-200c expression and breast cancer specific recurrence-free survival in PR – negative cancer cases. A) Kaplan-Meier analysis; B) Cox regression analysis including covariates age at diagnosis, nodal status, size of the tumor, histological type, *Her2*-status, and ER-status. OR (95% Cl), OR of breast cancer specific death with 95% Cl in multivariate analysis.

Next, we investigated whether miR-200c expression was associated with relapse-free-survival with the Kaplan-Meyer analysis. In the PR-negative group, patients with high miR-200c expression showed poor relapse-free survival in the 20-year follow-up compared to those with low miR-200c (*P* = 0.001, Figure 3A). The same result was obtained in the Cox regression analysis (*P* = 0.001, OR = 3.613, Figure 3B, table S5).

We also examined whether the miR-200c expression was related to local/distant recurrence of breast cancer. Among the PR-negative cases, we compared the miR-200c expression levels in patients with and without local/distant recurrence. The PR-negative cases (*n* = 72) with local or distant recurrence had higher miR-200c expression levels than those without recurrence (*P* = 0.006, OR = 3.965).

We then studied whether miR-200c expression was related to distant metastasis of breast carcinoma. Among the PR-negative cases, we compared the miR-200c expression levels in patients with and without distant metastasis. We found that cases with distant metastasis had higher miR-200c expression than cases without metastasis (*n* = 72, *P* = 0.015, OR = 3.390).

## Discussion

This study is the first to link breast cancer survival to miR-200c expression levels and PR status. Our data suggests that the PR status of the tumor influences whether miR-200c expression affects patient survival. We found that high miR-200c expression is an independent factor for predicting poor survival in the group of patients with PR-negative tumors. In contrast, we found that low miR-200c expression is an independent factor for predicting poor survival in the group of patients with PR-positive tumors. The same association was found in patients with tumors positive for both ER and PR. Among the PR-negative cases, high miR-200c expression was also associated with short recurrence-free survival. Among patients with PR-negative tumors, those that experienced local/distant recurrence had higher levels of miR-200c than the patients without recurrences. Similarly, among patients with PR-negative tumors, those that experienced distant metastases had higher miR-200c expression rates than the patients without metastases. These findings may facilitate the future diagnosis and management of patients with breast cancer.

ERs and PRs are key elements in breast cancer tumorigenesis, but microRNAs represent a relatively new chapter in the clinical setting of molecular laboratory research. Rather small number of studies has been published on the miR-200 family in connection with breast cancer and PR. Recently, the interactions between hormone receptors and miRNAs were found to contribute to breast cancer disease progression [34]. Several studies have shown that ER regulates miRNAs, but only a few studies have identified PR-regulated miRNAs. The connection between PR and miRNAs was first discovered in the genital tissue of women [35]–[39]. In another study, PR was found to regulate miRNAs in breast cancer. They showed that miR-16 and other miRNAs were down- or upregulated through synthetic progestin medroxyprogesterone acetate in breast cancer cell lines, but miR-200c was not one of them [27]. Further, as a response to progestin treatment, regulation of MiR-200c was altered in MCF10A cells [28] and a member of miR-200 family, miRNA-141 downregulated in T47D cells [30]. Again, PR expression was regulated by a progestin-upregulated miR-513a-5p [30] and progestins downregulated miR-29 in hormone receptor -positive breast cancer [31]. Also, PR-specific miRNAs have been identified. ER-specific downregulation of miR-181 and miR-26a targeting the 3′UTR of the PR sequence led to upregulation of the PR gene [29].

In the present study, it is possible that the PR modulated miR-200c expression. Indeed, miR-200c behaved differently in PR-negative and PR-positive cancers when analyzing the outcome of the patients. However, we did not observe significant differences in miR-200c expression levels between PR-negative and PR-positive cases in Kruskal-Wallis test when analyzing the status at the time of the diagnosis.

The PR is also regulated by estrogen, and the PR and ER tend to act in a consistent manner in breast cancer [40], [41]. In the present study, of the 94 PR-positive cases, 88 were also ER-positive. Nevertheless, when we included only the ER-positive cases in the calculations, no correlation was found between miR-200c expression and survival, despite the large number of patients and a relatively high rate of death from breast cancer (*P* = 0.259). Similarly, although the ER-negative group comprised fewer patients, they also showed no correlation between miR-200c expression and survival.

Nodal status is often one of the strongest predictor of the patient survival. The presence of nodes positive for cancer (node positivity) was statistically significant in the PR-positive group, when assessed with the multivariate analysis. However, in the PR-negative group, the Cox regression did not show significant node positivity in contrast to tumor size, histological type and ER status. This difference may have been due to the smaller number of samples with positive nodes in the PR-negative group, compared to that in the PR-positive group. On the other hand, PR positive tumors tend to be also ER positive that have a specific and efficient treatment. In these cases nodal status is often a strong prognostic factor. One might speculate that patients with PR negative cancers lacking an efficient treatment often have a poor prognosis and then nodal status would be of no relevance.

Previously, two studies have reported findings in line with those of the present study. [42], [43]. They also suggested that PR-status was a key factor in distinguishing among breast cancer subtypes. PR-positivity alone could predict whether patients with ER-positive breast cancer had a high risk (luminal B) or low risk (luminal A) prognosis [42], [43]. Notably, those studies did not include miR-200c expression. We did not compare outcomes between ER-positive cases with or without PR-positivity, because there were only a few ER-positive cases with PR-negativity. We found that the group with both ER-positivity and PR-positivity had the same outcome as the group with only PR-positivity. Our results indicated that, in addition to the PR status, the miR-200c expression level played a role in the subtype classification. Gene expression profiling studies have identified four significantly different classes of breast cancer, known as luminal A, luminal B, basal-like, and *Her2*-status positive [1], [2]. Our study included only 8 patients with luminal B class tumors and 100 patients with luminal A class tumors. We found no correlation between the miR-200c expression level and the luminal class. However, PR status was significantly associated to the outcome. Our study also included 29 triple negative cases, tumors negative for ER, PR and *Her2-*status, but no association with miR-200c expression among the cases was found. Therefore, a larger sample set that includes triple negative cancers is required for more accurate calculations.

Altered expressions of miRNAs have lately been linked with outcome of the cancer patients. High levels of miR-221 and miR-21 expression were associated with a poor prognosis in hepatocellular carcinoma [44], and high levels of miR-21 and miR-200c expression were associated with a poor survival in non-small-cell lung carcinoma [45]. In epithelial ovarian cancer, miR-200c expression was a potential predictor of survival and a biomarker for relapse [46]. In ovarian cancer, low miR-429 was associated with poor progression-free survival [47]. In pancreatic cancer, patients with high miR-200c levels displayed better survival than those with low miR-200c levels [48]. High miR-205 expression was linked to poor survival in endometrial cancer [49].

To our knowledge, miR-200c has not been previously linked to survival in breast cancer. However, it is plausible that low levels of miR-200c, which increase migration and invasion, might lead to poor survival. This hypothesis was consistent with our findings in the PR-positive group, but it was contradicted by our findings in the PR-negative group. Most previous studies have focused on ER-positive and PR-negative tumors. Although it would be interesting to study ER-negative and PR-positive tumors, it may be difficult, due to their rare occurrence.

Our results also underlined the effects of miR-200c expression and PR on recurrence-free survival. In the PR-negative group, patients with high miR-200c expression showed a shorter time to relapse than those with low miR-200c expression. This result was consistent with our finding that PR-negative cases had a worse outcome with high miR-200c expression than with low miR-200c expression. To our knowledge, this study was the first to link miR-200c expression to relapse rates in breast cancer.

Metaplastic breast cancers [14], [22] and claudin-low breast cancers [22] show reduced expression of the miR-200 family. In a study by Castilla et al., ER-positive tumors expressed high levels of miR-200 family and *Her2*-status positive, and triple negative tumors expressed intermediate levels of miR-200 family [22]. Our results differed from those of Castilla et al., possibly due to the small number of cases in our study for *Her2-*status positive and triple negative tumors. Cochrane et al. reported high levels of miR-200c in well-differentiated breast cell lines, but very low levels in poorly-differentiated cells lines [50]. In contrast to those results, we found an association between high miR-200c levels and poorly-differentiated grade 3 tumors, and this association remained significant after adjusting for PR status (*P* = 0.001 and *P* = 0.018 for PR positive cases and PR negative cases, respectively). However, it has to be taken into account that our material consisted of tumor samples that are more heterogenous than cancer cell lines. Bockmeyer et al. postulated that miR-200c and miR-429 were downregulated only in malignant myoepitheliomas, but not in basal-like breast cancers [51]. They suggested that high expression of miR-200c and miR-429 stabilized the epithelial phenotype and prevented invasive cell growth. Our findings of high miR-200c expression in grade 3 tumors contradicted Bockmeyer’s findings. The discrepancies between our results and Bockmeyer’s might be explained by differences in tumor samples. In general, patients with distant metastasis have a worse outcome than patients with no metastases. However, miR-200c was found to be overexpressed in distant metastasis compared to its expression in the primary tumor [52]. Our data showed high miR-200c expression in PR-negative tumors with patients that have distant metastasis and with gradus 3 patients in the whole material and in the PR negative patients. Although our results were originated in primary tumors these results are to some extend in line with the study of Gravgaard et al. It would be tempting to speculate that miR-200c expression increases during breast cancer progression and that it might be involved in metastatic process. However, we could not find further evidence when comparing miR-200c expression with tumor stage.

The transcriptional factors, ZEB1 and ZEB2 were previously demonstrated to be regulated by miR-200c, and they were found to be reciprocally expressed in breast cancer [12]–[14], [53]. We investigated the relationship between miR-200c and ZEB1, but found no correlation (data not shown). The data and procedure of the immunohistochemistry for ZEB1 was published previously [54]. However, this discrepancy may be explained by assessment of different phases of gene expression of ZEB1. Also, our tumor samples included both malignant and non malignant cells that may differ in miR-200c expression and ZEB1 protein expression. Thus, our results may present expression of miR-200c in the microenvironment of the epithelial tumor rather than only in malignant epithelial cells.

An *in silico* analysis with Targetscan [55] showed that *Cyp1b1* was a target for miR-200c. According to the KEGG pathway platform [56] *Cyp1b1* is also involved in steroid hormone biosynthesis. This connection suggested that miR-200c may play a role in steroid hormone biosynthesis at the gene level.

In conclusion, we have demonstrated that high miR-200c expression is an independent factor for predicting outcome for patients with invasive breast cancer. Furthermore, we found that high miR-200c expression was linked to grade 3 tumors. However, the nature of the prediction depended on the PR status of the tumor. In PR-negative tumors, high miR-200c expression was associated with a high probability of relapse, poor survival, and distant metastasis. Thus, increase of miR-200c expression could be a marker of breast cancer progression. Also, these results suggested a novel role for PR in breast cancer. In future, additional research with larger number of clinical samples will assure the role of miR-200c and PR in breast cancer risk, diagnostics and prognosis.

## Supporting Information

Figure S1 **MiR-200c expression and breast cancer specific survival in PR – and ER – positive cancer cases.**
(TIF)

Figure S2 **Kaplan-Meier analysis of miR-200c expression and breast cancer specific survival in PR - and ER – negative cancer cases.**
(TIF)

Table S1 Clinical characteristics of the tumor material. Abbreviations: *n*, number of cases.
(DOCX)

Table S2 Relative miR-200c expression values.
(DOCX)

Table S3 Multivariate analysis assessments of clinicopathological variables and miR-200c expression in breast ca specific survival with PR positive cancer cases. Abbreviations: *n*, number of cases; B coefficient with standard error (SE) from the multivariate analysis; Ref, reference category used for comparison. Note: clinical variables included: age at diagnosis, nodal status, tumor size, histological type, *Her2*-status and estrogen receptor status. ^a^: Low and high relative expression of miR-200c according to the median value.
(DOCX)

Table S4 Multivariate analysis assessments of clinicopathological variables and miR-200c expression in breast cancer specific survival with PR negative cancer cases. Abbreviations: *n*, number of cases; B coefficient with standard error (SE) from the multivariate analysis; Ref, reference category used for comparison. Note: clinical variables included: age at diagnosis, nodal status, tumor size, histological type, *Her-* status and estrogen receptor status. ^a^: Low and high relative expression of miR-200c according to the median value.
(DOCX)

Table S5 Multivariate analysis assessments of clinicopathological variables and miR-200c expression in breast cancer recurrence-free survival with PR negative cancer cases. Abbreviations: *n*, number of cases; B coefficient with standard error (SE) from the multivariate analysis; Ref, reference category used for comparison. Note: clinical variables included: age at diagnosis, nodal status, tumor size, histological type, *Her2*-status and estrogen receptor status. ^a^: Low and high relative expression of miR-200c according to the median value.
(DOCX)
