# Supplementary figures and images for: Overexpression of MicroRNA-200c Predicts Poor Outcome in Patients with PR-Negative Breast Cancer
Source: PLoS One. 2014 Oct 16;9(10):e109508. doi: 10.1371/journal.pone.0109508 (PMC4199599; doi:10.1371/journal.pone.0109508)

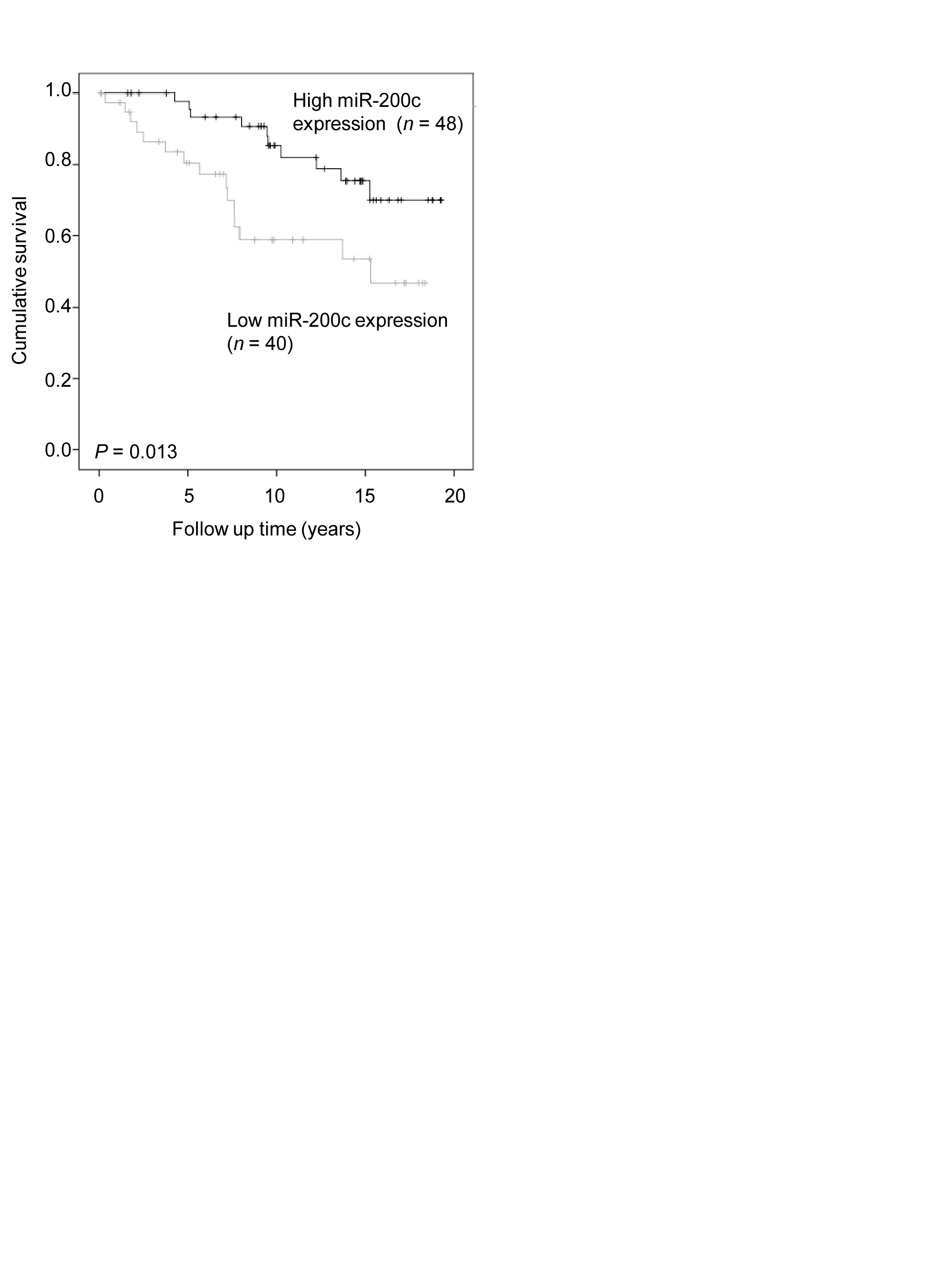

Supplement: Figure S1 — MiR-200c expression and breast cancer specific survival in PR – and ER – positive cancer cases. (TIF) [file pone.0109508.s001.tif]

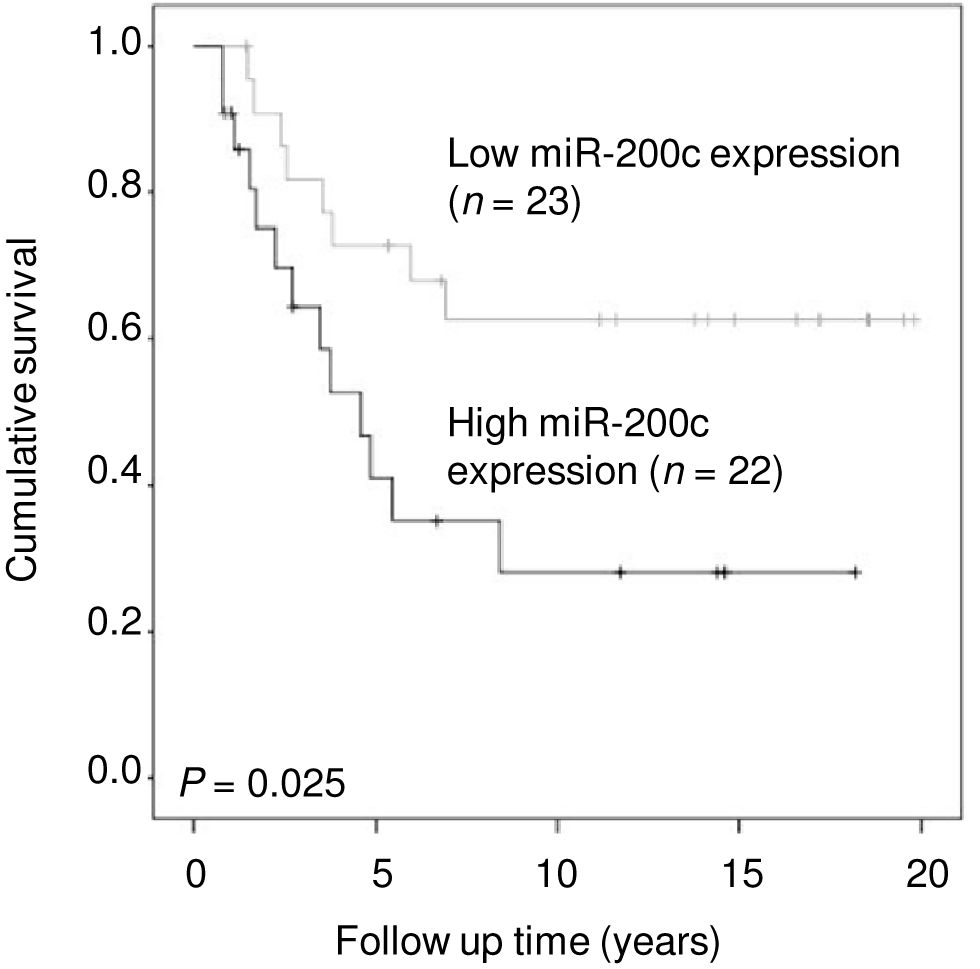

Supplement: Figure S2 — Kaplan-Meier analysis of miR-200c expression and breast cancer specific survival in PR - and ER – negative cancer cases. (TIF) [file pone.0109508.s002.tif]
