# Supplementary material for: Overexpression of MicroRNA-200c Predicts Poor Outcome in Patients with PR-Negative Breast Cancer
Source: PLoS One. 2014 Oct 16;9(10):e109508. doi: 10.1371/journal.pone.0109508 (PMC4199599; doi:10.1371/journal.pone.0109508)
Supplement: Table S1 — Clinical characteristics of the tumor material. Abbreviations: n, number of cases. (DOCX) [file pone.0109508.s003.docx]

**Table S1.** Clinical characteristics of the tumor material

| **Clinical variable** | ***n*** |
| --- | --- |
| Overall | 172 |
| Age at diagnosis |  |
| <= 59 | 85 |
| >=60 | 87 |
| Unknown | 0 |
| Patient status |  |
| Dead, breast cancer | 60 |
| Dead, other cause | 59 |
| Alive, no recurrence | 46 |
| Alive, recurrence | 7 |
| Unknown | 0 |
| Local/distant recurrence |  |
| Yes | 73 |
| No | 98 |
| Unknown | 1 |
| Histological grade |  |
| I and II | 106 |
| III | 61 |
| Unknown | 5 |
| Tumor stage |  |
| I | 44 |
| II | 100 |
| III and IV | 21 |
| Unknown | 7 |
| Histological type |  |
| Ductal | 117 |
| Lobular | 31 |
| Other | 19 |
| Unknown | 5 |
| Estrogen receptor |  |
| Negative | 51 |
| Positive | 115 |
| Unknown | 6 |
| Progesterone receptor |  |
| Negative | 72 |
| Positive | 94 |
| Unknown | 6 |
| *Her2*-status |  |
| Negative | 133 |
| Positive | 26 |
| Unknown | 13 |
| Triple negativity |  |
| Yes | 29 |
| No | 143 |
| Unknown | 0 |
| Luminal type A / B |  |
| Luminal A (er+, *_Her2*- and pr+) | 100 |
| Luminal B (er+, *Her2*+ and pr-) | 8 |
| Other / Unknown | 64 |
| Primary distant metastasis |  |
| Yes | 60 |
| No | 112 |
| Unknown | 0 |

Abbreviations: *n*, number of cases
