# Supplementary material for: Overexpression of MicroRNA-200c Predicts Poor Outcome in Patients with PR-Negative Breast Cancer
Source: PLoS One. 2014 Oct 16;9(10):e109508. doi: 10.1371/journal.pone.0109508 (PMC4199599; doi:10.1371/journal.pone.0109508)
Supplement: Table S2 — Relative miR-200c expression values. (DOCX) [file pone.0109508.s004.docx]

**Table S2.** Relative miR-200c expression values

| **Patient number** | **Fold change** |
| --- | --- |
| 1 | 0.042 |
| 2 | 0.095 |
| 3 | 0.113 |
| 4 | 1.305 |
| 5 | 0.044 |
| 6 | 0.097 |
| 7 | 0.288 |
| 8 | 7.561 |
| 9 | 0.488 |
| 10 | 0.322 |
| 11 | 0.342 |
| 12 | 0.749 |
| 13 | 2.642 |
| 14 | 0.318 |
| 15 | 0.267 |
| 16 | 9.213 |
| 17 | 0.476 |
| 18 | 0.811 |
| 19 | 0.385 |
| 20 | 1.122 |
| 21 | 0.111 |
| 22 | 0.206 |
| 23 | 1.082 |
| 24 | 2.845 |
| 25 | 0.118 |
| 26 | 0.054 |
| 27 | 0.166 |
| 28 | 0.155 |
| 29 | 0.165 |
| 30 | 0.166 |
| 31 | 0.098 |
| 32 | 0.195 |
| 33 | 0.132 |
| 34 | 0.091 |
| 35 | 0.165 |
| 36 | 0.184 |
| 37 | 0.183 |
| 38 | 0.113 |
| 39 | 0.155 |
| 40 | 0.181 |
| 41 | 1.994 |
| 42 | 0.369 |
| 43 | 0.238 |
| 44 | 0.830 |
| 45 | 0.538 |
| 46 | 0.689 |
| 47 | 1.325 |
| 48 | 1.870 |
| 49 | 3.287 |
| 50 | 0.772 |
| 51 | 0.237 |
| 52 | 1.549 |
| 53 | 0.380 |
| 54 | 0.372 |
| 55 | 0.145 |
| 56 | 0.604 |
| 57 | 0.265 |
| 58 | 0.442 |
| 59 | 0.147 |
| 60 | 0.130 |
| 61 | 0.358 |
| 62 | 0.149 |
| 63 | 0.894 |
| 64 | 0.384 |
| 65 | 0.272 |
| 66 | 0.166 |
| 67 | 0.160 |
| 68 | 0.469 |
| 69 | 0.517 |
| 70 | 0.287 |
| 71 | 0.207 |
| 72 | 0.265 |
| 73 | 0.493 |
| 74 | 0.020 |
| 75 | 0.109 |
| 76 | 0.122 |
| 77 | 0.048 |
| 78 | 0.091 |
| 79 | 0.667 |
| 80 | 0.175 |
| 81 | 0.108 |
| 82 | 0.097 |
| 83 | 0.091 |
| 84 | 0.039 |
| 85 | 0.105 |
| 86 | 0.090 |
| 87 | 0.000 |
| 88 | 4.279 |
| 89 | 0.091 |
| 90 | 0.075 |
| 91 | 0.259 |
| 92 | 3.946 |
| 93 calibrator | 1.016 |
| 94 | 0.064 |
| 95 | 0.083 |
| 96 | 0.077 |
| 97 | 0.059 |
| 98 | 0.080 |
| 99 | 0.108 |
| 100 | 0.006 |
| 101 | 0.121 |
| 102 | 0.135 |
| 103 | 0.095 |
| 104 | 0.062 |
| 105 | 2.589 |
| 106 | 0.083 |
| 107 | 0.133 |
| 108 | 0.054 |
| 109 | 0.254 |
| 110 | 24.190 |
| 111 | 0.380 |
| 112 | 0.405 |
| 113 | 0.943 |
| 114 | 2.195 |
| 115 | 2.978 |
| 116 | 0.444 |
| 117 | 0.313 |
| 118 | 0.800 |
| 119 | 0.360 |
| 120 | 0.268 |
| 121 | 0.155 |
| 122 | 0.171 |
| 123 | 0.580 |
| 124 | 0.706 |
| 125 | 0.133 |
| 126 | 0.175 |
| 127 | 0.039 |
| 128 | 0.253 |
| 129 | 0.142 |
| 130 | 0.095 |
| 131 | 0.183 |
| 132 | 0.121 |
| 133 | 0.168 |
| 134 | 0.114 |
| 135 | 0.187 |
| 136 | 0.626 |
| 137 | 0.224 |
| 138 | 0.221 |
| 139 | 0.327 |
| 140 | 0.184 |
| 141 | 0.582 |
| 142 | 0.604 |
| 143 | 0.432 |
| 144 | 0.452 |
| 145 | 0.465 |
| 146 | 0.011 |
| 147 | 0.242 |
| 148 | 0.336 |
| 149 | 0.372 |
| 150 | 0.391 |
| 151 | 1.472 |
| 152 | 1.183 |
| 153 | 0.523 |
| 154 | 0.495 |
| 155 | 0.812 |
| 156 | 0.065 |
| 157 | 1.703 |
| 158 | 0.695 |
| 159 | 0.759 |
| 160 | 0.645 |
| 161 | 0.957 |
| 162 | 0.781 |
| 163 | 0.509 |
| 164 | 0.355 |
| 165 | 0.752 |
| 166 | 1.638 |
| 167 | 0.664 |
| 168 | 2.787 |
| 169 | 0.407 |
| 170 | 0.506 |
| 171 | 0.300 |
| 172 | 0.744 |
| 173 | 0.824 |
