# Supplementary material for: Overexpression of MicroRNA-200c Predicts Poor Outcome in Patients with PR-Negative Breast Cancer
Source: PLoS One. 2014 Oct 16;9(10):e109508. doi: 10.1371/journal.pone.0109508 (PMC4199599; doi:10.1371/journal.pone.0109508)
Supplement: Table S3 — Multivariate analysis assessments of clinicopathological variables and miR-200c expression in breast ca specific survival with PR positive cancer cases. Abbreviations: n, number of cases; B coefficient with standard error (SE) from the multivariate analysis; Ref, reference category used for comparison. Note: clinical variables included: age at diagnosis, nodal status, tumor size, histological type, Her2-status and estrogen receptor status. a: Low and high relative expression of miR-200c according to the median value. (DOCX) [file pone.0109508.s005.docx]

**Table S3.** Multivariate analysis assessments of clinicopathological variables and miR-200c expression in breast ca specific survival with PR positive cancer cases

| **Clinical variable** | ***n*** | **B (SE)** | **Wald** | **OR (95% Cl)** | ***P*** |
| --- | --- | --- | --- | --- | --- |
| Age at diagnosis |  |  |  |  |  |
| <= 59 | 39 |  |  |  | 0.152 |
| >=60 | 47 |  | Ref. |  |  |
| Nodal status |  |  |  |  |  |
| Negative | 54 |  | Ref. |  |  |
| Positive | 32 | 1.04 (0.44) | 5.630 | 2.82 (1.20 – 6.62) | 0.018 |
| Tumor size |  |  |  |  | 0.462 |
| T1 | 36 |  | Ref. |  |  |
| T2 | 46 |  |  |  | 0.215 |
| T3 and T4 | 4 |  |  |  | 0.579 |
| miR-200c expression^a^ |  |  |  |  |  |
| Low | 37 | 1.43 (0.49) | 8.430 | 4.18 (1.59 – 10.96) | 0.004 |
| High | 49 |  | Ref. |  |  |
| Histological type |  |  |  |  | 0.107 |
| Ductal | 63 |  |  |  | 0.102 |
| Lobular | 14 |  |  |  | 0.039 |
| Other | 9 |  | Ref. |  |  |
| Estrogen receptor |  |  |  |  |  |
| Negative | 5 |  |  |  | 0.654 |
| Positive | 81 |  | Ref. |  |  |
| *Her2*-status |  |  |  |  |  |
| Negative | 79 |  | Ref. |  |  |
| Positive | 7 | - 2.37 (0.60) | 15.500 | 0.094 (0.030 – 0.30) | 0.000 |

Abbreviations: *n*, number of cases; B coefficient with standard error (SE) from the multivariate analysis; Ref, reference category used for comparison,

Note: clinical variables included: age at diagnosis, nodal status, tumor size, histological type, *Her2*-status and estrogen receptor status

^a^: Low and high relative expression of miR-200c according to the median value
