# Supplementary material for: Overexpression of MicroRNA-200c Predicts Poor Outcome in Patients with PR-Negative Breast Cancer
Source: PLoS One. 2014 Oct 16;9(10):e109508. doi: 10.1371/journal.pone.0109508 (PMC4199599; doi:10.1371/journal.pone.0109508)
Supplement: Table S4 — Multivariate analysis assessments of clinicopathological variables and miR-200c expression in breast cancer specific survival with PR negative cancer cases. Abbreviations: n, number of cases; B coefficient with standard error (SE) from the multivariate analysis; Ref, reference category used for comparison. Note: clinical variables included: age at diagnosis, nodal status, tumor size, histological type, Her- status and estrogen receptor status. a: Low and high relative expression of miR-200c according to the median value. (DOCX) [file pone.0109508.s006.docx]

**Table S4.** Multivariate analysis assessments of clinicopathological variables and miR-200c expression in breast cancer specific survival with PR negative cancer cases

| **Clinical variable** | ***n*** | **B (SE)** | **Wald** | **OR (95% Cl)** | ***P*** |
| --- | --- | --- | --- | --- | --- |
| Age at diagnosis |  |  |  |  |  |
| <= 59 | 37 |  |  |  | 0.856 |
| >=60 | 31 |  | Ref. |  |  |
| Nodal status |  |  |  |  |  |
| Negative | 36 |  | Ref. |  |  |
| Positive | 32 |  |  |  | 0.408 |
| Tumor size |  |  |  |  | 0.004 |
| T1 | 21 |  | Ref. |  |  |
| T2 | 36 | 0.042 (0.49) | 0.007 | 1.04 (0.40 – 2.71) | 0.932 |
| T3 and T4 | 11 | 1.66 (0.58) | 8.29 | 5.27 (1.70 – 16.32) | 0.004 |
| miR-200c expression^a^ |  |  |  |  |  |
| Low | 38 |  | Ref. |  |  |
| High | 30 | 1.23 (0.40) | 9.41 | 3.43 (1.56 – 7.55) | 0.002 |
| Histological type |  |  |  |  | 0.015 |
| Ductal | 48 | 2.11 (1.04) | 4.09 | 8.26 (1.07 – 63.93) | 0.043 |
| Lobular | 11 | 3.07 (1.13) | 7.37 | 21.61 (2.35 – 198.80) | 0.007 |
| Other | 9 |  | Ref. |  |  |
| Estrogen receptor |  |  |  |  |  |
| Negative | 43 | 0.88 (0.45) | 3.86 | 2.42 (1.00 – 5.83) | 0.049 |
| Positive | 25 |  | Ref. |  |  |
| *Her2*-status |  |  |  |  |  |
| Negative | 50 |  |  |  | 0.283 |
| Positive | 18 |  | Ref. |  |  |

Abbreviations: *n*, number of cases; B coefficient with standard error (SE) from the multivariate analysis; Ref, reference category used for comparison,

Note: clinical variables included: age at diagnosis, nodal status, tumor size, histological type, *Her-* status and estrogen receptor status

^a^: Low and high relative expression of miR-200c according to the median value
