# Supplementary material for: Overexpression of MicroRNA-200c Predicts Poor Outcome in Patients with PR-Negative Breast Cancer
Source: PLoS One. 2014 Oct 16;9(10):e109508. doi: 10.1371/journal.pone.0109508 (PMC4199599; doi:10.1371/journal.pone.0109508)
Supplement: Table S5 — Multivariate analysis assessments of clinicopathological variables and miR-200c expression in breast cancer recurrence-free survival with PR negative cancer cases. Abbreviations: n, number of cases; B coefficient with standard error (SE) from the multivariate analysis; Ref, reference category used for comparison. Note: clinical variables included: age at diagnosis, nodal status, tumor size, histological type, Her2-status and estrogen receptor status. a: Low and high relative expression of miR-200c according to the median value. (DOCX) [file pone.0109508.s007.docx]

**Table S5.** Multivariate analysis assessments of clinicopathological variables and miR-200c expression in breast cancer recurrence-free survival with PR negative cancer cases

| **Clinical variable** | ***n*** | **B (SE)** | **Wald** | **OR (95% Cl)** | ***P*** |
| --- | --- | --- | --- | --- | --- |
| Age at diagnosis |  |  |  |  |  |
| <= 59 | 37 |  |  |  | 0.609 |
| >=60 | 31 |  | Ref. |  |  |
| Nodal status |  |  |  |  | 0.371 |
| Negative | 36 |  | Ref. |  |  |
| Positive | 32 |  |  |  |  |
| Tumor size |  |  |  |  | 0.013 |
| T1 | 21 |  | Ref. |  |  |
| T2 | 36 | 0.037 (0.46) | 0.006 | 1.04 (0.42 – 2.55) | 0.936 |
| T3 and T4 | 11 | 1.39 (0.56) | 6.17 | 4.01 (1.34 – 12.02) | 0.013 |
| miR-200c expression^a^ |  |  |  |  |  |
| Low | 38 |  | Ref. |  |  |
| High | 30 | 1.29 (0.39) | 10.85 | 3.61 (1.68 – 7.76) | 0.001 |
| Histological type |  |  |  |  | 0.088 |
| Ductal | 48 | 2.03 (1.04) | 3.84 | 7.62 (1.00 – 58.04) | 0.050 |
| Lobular | 11 | 2.42 (1.10) | 4.84 | 11.20 (1.30 – 96.33) | 0.028 |
| Other | 9 |  | Ref. |  |  |
| Estrogen receptor |  |  |  |  | 0.155 |
| Negative | 43 |  |  |  |  |
| Positive | 25 |  | Ref. |  |  |
| *Her2*-status |  |  |  |  | 0.992 |
| Negative | 50 |  |  |  |  |
| Positive | 18 |  | Ref. |  |  |

Abbreviations: *n*, number of cases; B coefficient with standard error (SE) from the multivariate analysis; Ref, reference category used for comparison,

Note: clinical variables included: age at diagnosis, nodal status, tumor size, histological type, *Her2*-status and estrogen receptor status

^a^: Low and high relative expression of miR-200c according to the median value
